# *In Vitro* and* In Vivo* Quality Evaluation of Glibenclamide Tablets Marketed in Addis Ababa, Ethiopia

**DOI:** 10.1155/2018/7916368

**Published:** 2018-07-18

**Authors:** Haile Kassahun, Kaleab Asres, Ayenew Ashenef

**Affiliations:** ^1^Department of Pharmacy, College of Health Sciences, Wollo University, P.O. Box. 1145, Dessie, Ethiopia; ^2^Department of Pharmaceutical Chemistry and Pharmacognosy, School of Pharmacy, College of Health Sciences, Addis Ababa University, P.O. Box. 1176, Addis Ababa, Ethiopia

## Abstract

Good quality drugs fulfilling the regulatory parameters and produced per the current good manufacturing (CGMP) standards are very critical for best therapeutic outcome in patient therapy. Hence, this study assesses quality as well as physicochemical bioequivalence of five brands of glibenclamide tablets marketed in Addis Ababa using* in vitro* and* in vivo* methods. Friability, disintegration, dissolution, and assay for the content of active ingredients were evaluated using the methods described in the British Pharmacopeia (2009) and United States Pharmacopeia (2007). All the brands of glibenclamide tablets complied with the official specification for hardness, friability, disintegration, and assay. Difference factor (f1) values were less than 15 and similarity factor (f2) values were greater than 50 for all products of glibenclamide. The hypoglycemic effect of different products of glibenclamide tablets was evaluated on normoglycemic mice. The* in vivo* studies indicated that there is no significant difference in percent reduction of blood glucose level between the brands of glibenclamide and the innovator product (p > 0.05). Hence, based on the* in vivo* results and* in vitro* dissolution studies, the brands might be substituted with the innovator product in clinical practice.

## 1. Introduction

Good quality medicines are a prerequisite for a successful treatment. Drug quality is currently receiving a growing international attention. Over the past decade, there has been an increase in public awareness of the existence of counterfeit and substandard drugs which have been increasingly reported in developing countries where drug regulations are less effective or totally absent [1]. Use of poor quality products may lead to therapeutic failure, increased morbidity and mortality, erosion of public confidence in health care, unexpected side effects, and antimicrobial resistance [2].

The introduction of generic drug products from multiple sources into the health care delivery system of many developing countries has been accompanied by a variety of problems of which the most critical is the widespread distribution of fake and substandard drug products [3]. To assist in substitution of branded innovator product with generics for affordability and at the same time to achieve therapeutic efficacy, bioequivalence studies become important [4]. Bioequivalence studies for generic products are essential to ensure the absence of any significant difference in the rate and extent to which the active ingredients become available at the site of drug action administered under similar route and conditions [5]. Generic drug products must satisfy the same standards of quality, efficacy, and safety as those applicable to the innovator product [6]. Bioavailability or bioequivalence studies may involve both* in vivo *and* in vitro *studies [7].

Glibenclamide (also known as glyburide) is an oral hypoglycemic agent of the sulphonylurea group widely used in the treatment of type II diabetes mellitus (DM) [8]. It lowers blood glucose concentration by stimulating the release of insulin from the pancreatic beta cells [9]. Globally, there are several generics of glibenclamide tablets available within the drug delivery system after the expiration of patent on the innovator brand. Studies that deal about comparative* in vitro* quality evaluation of generics of glibenclamide tablets of different countries have been published. El-Sabawi* et al*. [10] studied five generics of glibenclamide tablets available in Jordan market and reported that they exhibited dissolution profiles that are significantly different from each other and from that of the original Daonil. In addition, Elhamili* et al*. [11] evaluated three brands of glibenclamide tablets available in Libyan market and reported that all products were within the British Pharmacopeia (BP) specifications.

The increasing use of glibenclamide tablets in clinical practice necessitates the need to monitor and ascertain the quality of the various brands available in the drug market. Oral glibenclamide is widely used in Ethiopia with several new brands introduced into the Ethiopian market in recent years. Variety of drugs in circulation often put clinicians and pharmacists into difficult situation of choice for the possibility of interchangeability among brands [12, 13]. Use of substandard products may lead to poor blood glucose control, thus causing life threatening complications. Despite the widespread presence of non-insulin dependent diabetes mellitus (NIDDM) in Ethiopia and extensive use of glibenclamide, there are no reports on the bioavailability and bioequivalence of the various brands in the country. Hence, the present study was carried out to assess the quality and physicochemical bioequivalence of glibenclamide tablets available in Addis Ababa, Ethiopian market.

## 2. Materials and Methods

### 2.1. Materials

Different brands of 5 mg glibenclamide tablets were bought from various pharmacy retail outlets in Addis Ababa, Capital city of Ethiopia. All the brands used were within their shelf life at the time of study. The detailed descriptions for these products are presented in Table 1. Standard glibenclamide was obtained from Ethiopian Food, Medicines and Health care Administration and Control Authority (EFMHACA) and is of United States Pharmacopoeia (USP) reference standard. All chemicals used were of analytical grade.

### 2.2. Reagents and Solvents

The chemicals and reagents used to perform the experiments were the following: monobasic ammonium phosphate (FARMITALIA CAROERBA, Italy), sodium hydroxide (BDH limited, Poole, England), HPLC grade acetonitrile (BDH Laboratory Supplies, England), 85% phosphoric acid (Riedel-de Haen, Germany), potassium phosphate monobasic (Fisher Scientific, USA), HPLC grade methanol (Park Scientific Limited, UK), and distilled water.

### 2.3. Instruments/Equipment

The following instruments were used for the experiments: analytical balance (Mettler Toledo, Switzerland), hardness tester (Schleuniger, 2E/205, Switzerland), friability tester (ERWEKA, TAR 20, Germany), disintegration apparatus (CALEVA, G.B. Caleva Ltd., UK), dissolution apparatus (ERWEKA, DT600, Germany), UV-Visible Spectrophotometer (Single beam Spectrophotometer, CM2203, Belarus), filter paper (diameter 110, lot ER0692-1, Schleicher and Schell, Germany), Vacuum filter (Scientific Laboratories Supplies, Germany), glucometer (Prodigy Diabetes care LLC, USA), PH meter (Mettler Toledo, Switzerland), and HPLC-UV (Shimadzu Corporation, C_18_ stainless steel column (15 cm x10 mm), UV-VIS detector, Japan).

### 2.4. Experimental Animals

Swiss albino mice of either sex weighing 21- 30 g and age 5-6 weeks were obtained from the Department of Biology, Addis Ababa University. All animals were housed in an air-conditioned room. They were offered standard pellets and water* ad libitum.* The animals were conditioned one week prior to the experiments.

### 2.5. Methods

Drug quality assessment experiments were done using pharmacopeial procedures described in the USP/NF XXIV, 2000 [14], USP/NF 25, 2007 [15], and BP, 2009 [16].

#### 2.5.1. Hardness Test

The hardness of each tablet was determined by selecting six tablets randomly using a hardness tester. Each tablet was placed between two anvils and force was applied to the anvils, and the crushing strength that causes the tablet to break was recorded. Crushing strength of average of six tablets was recorded.

#### 2.5.2. Friability Test

Ten tablets from each brand were weighed using an analytical balance. Tablets were placed in the drum of the friability tester and subjected to rotation at 25 revolutions per minute (rpm) for four minutes (100 times). Then, tablets were dedusted and weighed. The weights were compared with their initial weights and then percentage friability was calculated based on the weight difference obtained.

#### 2.5.3. Disintegration Time

Disintegration time test is carried out according to USP/NF (2007) specification. Six tablets were placed in a disintegration tester filled with distilled water at 37±0.5°C. The tablets were considered as completely disintegrated when all the particles have passed through the wire mesh. This time was recorded in minutes as disintegration time.

#### 2.5.4. Chemical Assay

Assay of glibenclamide was done using BP (2009) method by high pressure liquid chromatography (HPLC) equipped with UV/VIS detector, a C_18_ stainless steel column (15 cm x10 mm). The mobile phase composition was a mixture of acetonitrile and 1.36 % w/v solution of potassium dihydrogen orthophosphate (previously adjusted to p^H^ 3.0 with 85 % orthophosphoric acid) in a ratio of 47:53, with flow rate of 1.5 ml/min. The sample injection volume was 20 *μ*l and the wavelength was set at 300 nm.

*Sample Preparation. *Twenty tablets were weighed and powdered. A quantity of the powdered tablets containing 5 mg of glibenclamide was mixed with a mixture of 2 ml of wate*r *and 20 ml of methanol with the aid of ultrasonic bath and filtered through a vacuum filter.

*Standard Preparation. *Glibenclamide working standard (50 mg) was dissolved in 10 ml of methanol with the aid of ultrasonic bath for 20 min, sufficient methanol was added to produce 50 ml of stock solution and finally the resulting solution was diluted to 200 ml with methanol. From the stock solution, serial dilutions were made to obtain calibration concentrations of 181.8181 *μ*g/ml, 204.5454 *μ*g/ml, 227.2727 *μ*g/ml, 250 *μ*g/ml, and 272.7272 *μ*g/ml. Equal volumes (20 *μ*l) of the standard preparation and the assay preparation were separately injected into the HPLC system and then the chromatograms were recorded and the peak areas were obtained to be employed for amount calculation.

#### 2.5.5. Dissolution Test

The dissolution of glibenclamide tablets was done according to the specification of USP/NF XXIV, 2000 [14] using dissolution apparatus type II (paddle apparatus) with the rate of 50 rpm at 37±0.5°C on six tablets of each brand. 10 ml sample was withdrawn at 10, 20, 30, 45, and 60 min and an equivalent amount of fresh dissolution medium was replaced. Filtered samples were then appropriately diluted and absorbance readings were taken with UV/Visible Spectrophotometer at wavelength of 226 nm. The concentration of each sample was determined from calibration curve. The percent of drug release at each time was calculated.

*Standard Preparation. *Stock solution of 100 *μ*g/ml was prepared by dissolving glibenclamide working standard (50 mg) in 500 ml of phosphate buffer p^H^ 7.4. 10 ml of the resulting solution was diluted with phosphate buffer to 100 ml to obtain 0.01 mg/ml of the working standard solution. Finally, from the resulting solution, 16, 20, 24, 28, 32, and 36 ml were pipetted out separately into 50 ml volumetric flask and was made to volumes to get a concentration range of 0.0032, 0.004, 0.0048, 0.0056, 0.0064, and 0.0072 mg/ml, respectively. The absorbance was measured at 226 nm using UV-Visible Spectrophotometer.

#### 2.5.6. Test for Hypoglycemic Effect

Different studies on antidiabetic activity of plant extracts in normoglycemic rats have been conducted [17, 18] and in the present study, the method described by Saidu* et al.* [17] was followed to study the hypoglycemic effect of products of glibenclamide on normoglycemic mice.

Swiss albino mice of either sex were randomly housed in stainless steel cages and were offered standard pellets with drinking water* ad libitum. *The animals were acclimatized for one week prior to the experiments. Then, the animals were divided into 6 groups of five mice each. Groups (1-5) were treated with different brands of glibenclamide tablets (5 mg/kg p.o). Animals in group 6 received 90 % Dimethyl Sulfoxide (DMSO) as a control. All groups were subjected to fasting for 16 h before the experiment. Powdered glibenclamide tablets were dissolved in DMSO for oral administration. Blood samples were collected at 0, 1, 2, 3, and 4 h from each group by cutting the tail tip of the mice, then blood glucose level was measured using glucometer [19, 20].

### 2.6. Data Analysis

Data obtained was treated using ORIGIN® graphing and scientific analysis software program, Microsoft Excel 2007 and Windows SPSS Version 20. Comparison and statistical significance was determined by one-way analysis of variance (ANOVA). All data were analyzed at a 95 % confidence interval (P < 0.05).

## 3. Results and Discussions

### 3.1. Hardness and Friability Test

The mean hardness value of glibenclamide tablets is shown in Table 2. The results showed that the brands had mean hardness value within the range of 50.3±2.65-101.66±3.38 N. Melix had the highest hardness value (101.66±3.38 N) and Glamide had the lowest hardness value (50.3±2.65 N). A force of about 40 N is the minimum requirement for satisfactory tablet hardness [21]. Hence, the tablets of all brands of glibenclamide had satisfactory hardness. Similarly, the percent weight loss of the tablets after friability test is shown in Table 2. The results showed that all the brands of glibenclamide tablets had friability values ranging from 0.105-0.106%. The lowest and highest friability have been obtained for Glitisol (0.105) and Glamide (0.289). According to USP (2007), percent friability value of tablets should be less than 1%. Thus, all brands passed the friability specification.

Hardness or crushing strength of tablets is an important parameter which helps to assess the resistance of the tablet to breakage under condition of storage, transportation, and handling [22]. It is, therefore, important that tablets are of optimum hardness [23]. Friability test is closely related to tablet hardness and is designed to evaluate the ability of the tablet to withstand abrasion in coating, packaging, handling, and transporting and other manufacturing processes [24]. Generally, adequate tablet hardness as well as reasonable friability is required for consumer acceptance [25].

### 3.2. Disintegration Test

The mean disintegration time for the different brands of glibenclamide tablets is shown in Table 2. The results showed that all brands passed the disintegration test according to USP (2007), which specifies 30 min for uncoated and film coated tablets. All products of glibenclamide tablets had mean disintegration time of less than 5 min except Glitisol. The rapid disintegration time exhibited by all the brands might be due to type and amount of disintegrant used in the formulation. Glitisol showed the longest disintegration time (6.44±0.44) which correlates with its low friability and high hardness values.

Tablet disintegration is a prerequisite to dissolution and subsequent absorption of the drug from its dosage form. A drug incorporated in a tablet will be released rapidly as the tablet disintegrates. The rate of disintegration affects the dissolution and subsequently the therapeutic efficacy of the medicine [12]. Different formulation factors are known to affect results in disintegration test. The type and amount of excipients used in tablet formulation as well as the manufacturing process are all known to affect both the disintegration and dissolution parameters [26].

### 3.3. Chemical Assay

**Figure 1:**
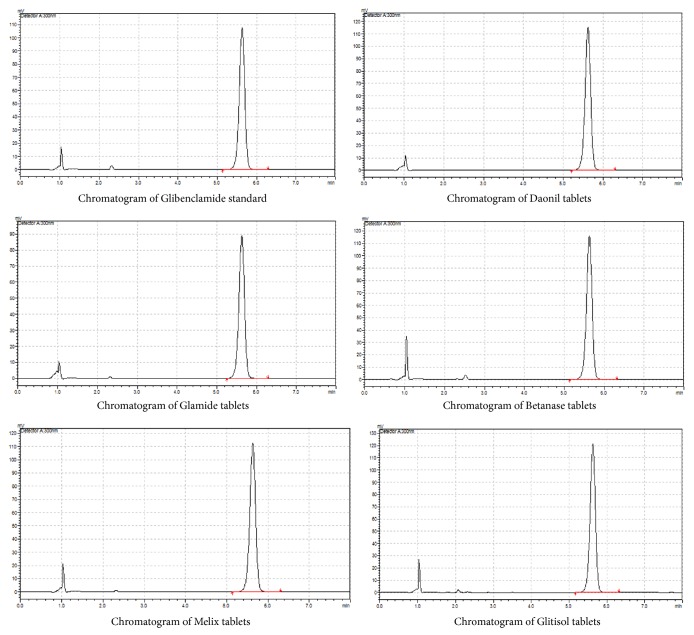
Typical HPLC chromatogram's obtained in the assay.

The assay determines the concentration of the active pharmaceutical ingredient in a sample. The assay was done by using HPLC. Samples were injected and the averages of the peak areas were calculated. Representative peaks are shown in Figure 1.

The results for the mean percentage label claim of the different brands of glibenclamide tablets are depicted in Table 2. The products were assayed according to the method outlined in BP (BP, 2009). BP (2009) states glibenclamide tablets should contain not less than 95.0 % and not more than 105.0 % of the stated amount. As indicated in Table 2, all products fulfilled the pharmacopeial standards for percentage content of active ingredient.

The highest percentage content was obtained for Glitisol (104.33 %), while the least drug content was obtained for Daonil (95.53 %). Tablets contain specific amount of active ingredient with allowable variable limit and assay of tablet ensures the amount of active ingredient which is indicative of its efficacy and stability of the product. Statistical comparison for drug content indicates that with 95 % confidence interval, there is significant difference in the drug content between Daonil and all other brands of glibenclamide (P<0.05).

### 3.4. Dissolution Test

**Figure 2:**
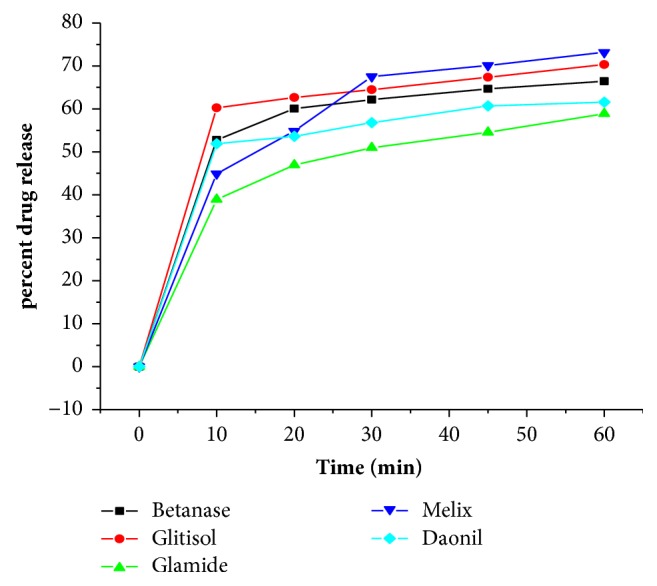
Dissolution profiles of different products of glibenclamide tablets.

Five different products of glibenclamide tablets were studied, with Daonil being the innovator. To compare the dissolution profiles, dissolution curve (based on mean percentages of drug released) of test and innovator products were combined and depicted in Table 3 and Figure 2.

From Figure 2, it can be seen that all products, including the innovator product (Daonil), did not release significant percentage of the drug within the first 30 min. In fact, Betanase, Glitisol, and Melix released more than 60 % drug within 30 min and Glamide and Daonil released about 50 % drug within 30 min.

Similarity factor (*f*2) and difference factor (f1), model independent parameters, derived from the dissolution profiles were calculated to compare dissolution profiles of the different products of glibenclamide tablets [27].

(1) f1=∑t=1nRt−Tt∑t=1nRt×100,

(2) f2=50×log10⁡1001+∑t=1nRt−Tt2/n,

where Rt and Tt are the cumulative percentage of dissolved drug for the reference and test formulation at time t, respectively, and n is the number of time points. According to these guides, generally, f1 values up to 15 (0–15) and f2 values greater than 50 (50–100) ensure similarity or equivalence of the two dissolution profiles [28]. f1 and f2 values were calculated between the test products and reference product (Daonil) and are illustrated in Table 4.

As shown in Table 4, the f1 values are found to be less than 15 and f2 values are greater than 50 for all products of glibenclamide which shows similarity or equivalence of the dissolution profiles. Therefore, this confirmed similarity between all products of glibenclamide compared with the innovator product (Daonil) and indicated that the release of glibenclamide from all products was similar to the reference.

### 3.5. *In Vivo* Studies

#### 3.5.1. Hypoglycemic Effect of Glibenclamide Tablets on Normoglycemic Mice

Quantification of pharmacologic effect is one possible way to assess a drug's bioavailability. This method is based on the assumption that a given intensity of response is associated with a particular drug concentration at the site of action [7].

T_max_ after oral administration of glibenclamide in mice is 2 h [29]. As shown in Table 5, Glamide (45.99 %) showed highest percent reduction in blood glucose level at T_max_ while the least was observed for Betanase with 36.27 % at T_max_ (2 h).

Statistical analysis was conducted using Dunnett's t-test and it was found that, at 95% confidence interval, there was no significant difference in the percent reduction of blood glucose level between Daonil and Glitisol, between Daonil and Melix, and between Daonil and Betanase at l h (p >0.05), while Glamide was significantly different from the innovator product. Glamide showed highest reduction in blood glucose level compared to the innovator product. At 2 h (T_max_ value) and 4 h, there was no significant difference between Daonil and all the other brands of glibenclamide (p >0.05), suggesting that the serum blood glucose profiles generated by the reference tablets were comparable to those produced by the test products. Hence, based on* in vivo* results and* in vitro* dissolution studies, the brands might be substituted with the innovator product in clinical practice.

From both* in vitro* and* in vivo *studies, it was shown that there are minor variations among the generic products of glibenclamide tablets. Despite that, all the studied glibenclamide tablets distributed in the Ethiopian market are of good quality products. This might be as a result of strict adherence to good manufacturing practice in the process of manufacturing these tablets, effective control activities of EFMHACA, and the proper storage of these drugs by wholesalers, retailers, and pharmacies.

## 4. Conclusion

This study assessed quality as well as physicochemical bioequivalence of five brands of glibenclamide tablets marketed in Addis Ababa using* in vitro *and* in vivo* methods. The study confirmed that brands of glibenclamide tablets complied with the official specification for hardness, friability, assay, and disintegration. The f1 values were less than 15 and f2 values were greater than 50 for all products of glibenclamide. This suggests that release of the drug from all products of glibenclamide is similar to the innovator product. In addition,* In vivo* studies of the products of glibenclamide tablets indicated that there is no significant difference in percent reduction of blood glucose level between Daonil and the other brands (p > 0.05). Hence, based on the* in vivo* results and* in vitro* dissolution studies, any of the glibenclamide products might be substituted with the innovator product in clinical practice.

## Figures and Tables

**Table 1:** Detailed description of products of glibenclamide tablets included in the study.

Brand	Manufacturer	Batch Number	Mfg. date	Exp. date
Betanase	Cadila Health Care Limited (India)	GM3429	12/2012	11/2016
Daonil	Sanofi Aventis (France)	3AP9A	03/2013	03/2015
Glamide	Cadila Pharmaceuticals PLC (Ethiopia)	D12010BX75	09/2012	08/2016
Glitisol	Remedica Ltd. (Cyprus)	52994	10/2012	10/2017
Melix	Lagap SA ( Switzerland)	B050	05/2011	04/2016

**Table 2:** Results of hardness, friability, disintegration tests, and chemical assay of different brands of glibenclamide tablets included in the study.

Drug Product	Brand	Hardness (N) ±SD	Friability (%)	Disintegration time ±SD (Min)	Assay (%W/W ±SD)
Glibenclamide 5 mg	Betanase	65.83±6.79	0.224	2.83±0.25	101.05±0.16
	Daonil	81.66±4.5	0.106	1.43±0.14	95.53±0.54
	Glamide	50.3±2.65	0.289	1.36±0.09	100.68±1.83
	Glitisol	85.5±3.39	0.105	6.44±0.44	104.33±0.04
	Melix	101.66±3.38	0.126	2.29±0.19	97.32±0.65

**Table 3:** Time dependent drug release of different products of glibenclamide tablets.

sampling time (min)	Percent of drug release (W/W) ±RSD				
	Betanase	Glitisol	Glamide	Melix	Daonil
10	52.77± 0.18	60.25±0.031	38.95±0.09	44.94±0.04	51.89±0.028
20	60.06±0.003	62.67±0.032	46.93±0.2	54.85±0.006	53.64±0.012
30	62.18 ±0.02	64.5±0.018	50.99±0.1	67.5 ±0.067	56.79± 0.06
45	64.65 ±0.28	67.358±0.01	54.57±0.07	70.13± 0.04	60.698±0.02
60	66.45± 0.01	70.34±0.019	58.88±0.08	73.16±0.035	61.57 ±0.072

**Table 4:** Similarity and difference factors of glibenclamide products.

Products	Betanase	Glamide	Glitisol	Melix
f1	7.56	12.04	14.24	14.02
f2	65.912	55.695	54.26	52.61

**Table 5:** Percent reduction in blood glucose level of normoglycemic mice for glibenclamide products.

Brand	Percent reduction in blood glucose level (mg/dl) ± SD			
	1 h	2 h	3 h	4 h
Betanase	31.55±9.52	36.27±14.69	30.15±6.07	30.4±16.56
Daonil	17.14±10.98	40.58±11.65	50.71±9.54	33.81±18
Glamide	43.16±13.87	45.99±20.65	46.7±15.43	50.38±7.4
Glitisol	40.57±10.9	44.62±9.48	47.53±5.81	52.22±3.28
Melix	33.93±23.33	38.77±11.16	37.92±7	37.29±8.83

## Data Availability

The data used to support the findings of this study are included within the article.
